# HPC-CLUST: distributed hierarchical clustering for large sets of nucleotide sequences

**DOI:** 10.1093/bioinformatics/btt657

**Published:** 2013-11-09

**Authors:** João F. Matias Rodrigues, Christian von Mering

**Affiliations:** Institute of Molecular Life Sciences and Swiss Institute of Bioinformatics, University of Zurich, Zurich, Switzerland

## Abstract

**Motivation: **Nucleotide sequence data are being produced at an ever increasing rate. Clustering such sequences by similarity is often an essential first step in their analysis—intended to reduce redundancy, define gene families or suggest taxonomic units. Exact clustering algorithms, such as hierarchical clustering, scale relatively poorly in terms of run time and memory usage, yet they are desirable because heuristic shortcuts taken during clustering might have unintended consequences in later analysis steps.

**Results: **Here we present HPC-CLUST, a highly optimized software pipeline that can cluster large numbers of pre-aligned DNA sequences by running on distributed computing hardware. It allocates both memory and computing resources efficiently, and can process more than a million sequences in a few hours on a small cluster.

**Availability and implementation: **Source code and binaries are freely available at http://meringlab.org/software/hpc-clust/; the pipeline is implemented in C++ and uses the Message Passing Interface (MPI) standard for distributed computing.

**Contact: **mering@imls.uzh.ch

**Supplementary Information: **Supplementary data are available at *Bioinformatics* online.

## 1 INTRODUCTION

The time complexity of hierarchical clustering algorithms (HCA) is quadratic 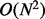 or even worse 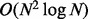, depending on the selected cluster linkage method (Day and Edelsbrunner, 1984). However, HCAs have a number of advantages that make them attractive for applications in biology: (i) they are well defined and should be reproducible across implementations, (ii) they require nothing but a pairwise distance matrix as input and (iii) they are agglomerative, meaning that sets of clusters at arbitrary similarity thresholds can be extracted quickly by post-processing, once a complete clustering run has been executed. Consequently, HCAs have been widely adopted in biology, in areas ranging from data mining to sequence analysis to evolutionary biology.

Apart from generic implementations, a number of hierarchical clustering implementations exist that focus on biological sequence data, taking advantage of the fact that distances between sequences can be computed relatively cheaply, even in a transient fashion. However, the existing implementations such as MOTHUR (Schloss *et al.*, 2009), ESPRIT (Sun *et al.*, 2009) or RDP online clustering (Cole *et al.*, 2009), all struggle with large sets of sequences. In light of these performance limits, heuristic optimizations have also been implemented such as CD-HIT (Li and Godzik, 2006) and UCLUST (Edgar, 2010).

Hierarchical clustering starts by considering every sequence separately and merging the two closest ones into a cluster. Then, iteratively, larger clusters are formed, by joining the closest sequences and/or clusters. The distance between two clusters with several sequences will depend on the clustering linkage chosen. In single linkage, it is the similarity between the two most similar sequences; in complete linkage, between the two most dissimilar sequences; and in average linkage, the average of all pairwise similarities. The latter method is also known as the Unweighted Pair Group Method with Arithmetic Mean (UPGMA) and is often used in the construction of phylogenetic guide trees.

In the type of approach used by CD-HIT and UCLUST, each input sequence is considered sequentially, and is either added to an existing cluster (if it is found to meet the clustering threshold) or is used as a seed to start a new cluster. Although this approach is extremely efficient, it can lead to some undesired characteristics (Sun *et al.*, 2012): (i) it will create clusters with sequences that may be more dissimilar than the chosen clustering threshold; (ii) it can occur that a new cluster is created close to an existing cluster, but at a distance just slightly longer than the clustering threshold; at this point, any new sequences close to both clusters will be split among the two clusters, whereas previous sequences will have been added to only the first cluster; this effectively results in a reduction of the clustering threshold locally; and (iii) different sequence input orders will result in different sets of clusters due to different choices of the seed sequences. Point (i) also affects HCA using single linkage and to a lesser extent average linkage, but does not occur with complete linkage.

Here we present a distributed implementation of an HCA that can handle large numbers of sequences. It can compute single-, complete- and average-linkage clusters in a single run and produces a merge-log from which clusters can subsequently be parsed at any threshold. In contrast to CD-HIT, UCLUST and ESPRIT, which all take unaligned sequence data as their input, HPC-CLUST (like MOTHUR) takes as input a set of pre-aligned sequences. This allows for flexibility in the choice of alignment algorithm; a future version of HPC-CLUST may include the alignment step as well. For further details on implementation and algorithms, see the Supplementary Material.

## 2 METHODS

For all benchmarks, we used one or more dedicated Dell Blade M605 compute nodes with 2 quad-core Opteron 2.33 GHz processors and 24 GB of random access memory. The most recent version of each software pipeline was used: HPC-CLUST (v1.0.0), MOTHUR (v.1.29.2), ESPRIT (Feb. 2011), CD-HIT (v4.6.1) and UCLUST (v6.0.307). Detailed information on settings and parameters is available in the Supplementary Material.

We compiled a dataset of publicly available full-length 16S bacterial ribosomal RNA sequences from NCBI Genbank. Sequences were aligned using INFERNAL v1.0.2 with a 16S model for bacteria from the ssu-align package (Nawrocki *et al.*, 2009). Importantly, INFERNAL uses a profile alignment strategy that scales linearly *O*(*N*) with the number of sequences, and can be trivially parallelized. Indels were removed and sequences were trimmed between two well-conserved alignment columns, such that all sequences had the same aligned length. The final dataset consisted of 1 105 195 bacterial sequences (833 013 unique) of 1301 in aligned length.

## 3 RESULTS

### 3.1 Clustering performance on a single computer

**Fig. 1.**
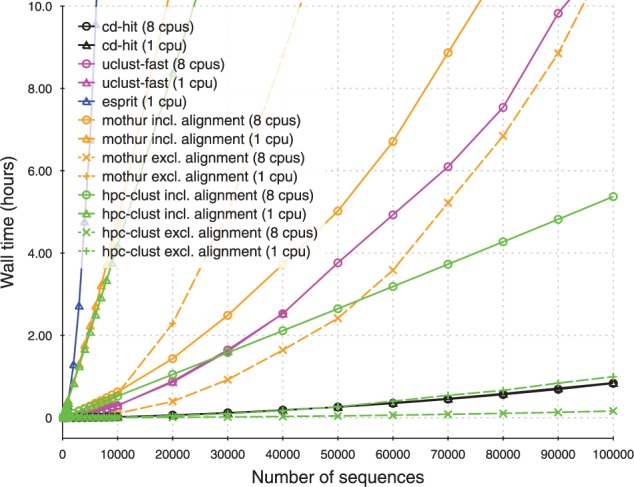
Runtime comparisons. For HPC-CLUST and MOTHUR, runtimes are shown both including and excluding sequence alignment runtime. UCLUST and CD-HIT exhibited only negligible decreases in runtime when using multiple threads. Identity threshold for clustering was 98% identity

HPC-CLUST has been highly optimized for computation speed and memory efficiency. It is by far the fastest of the exact clustering implementations tested here, even when running on a single computer (Fig. 1). Compared with MOTHUR, it produces identical or nearly identical clustering results (see Supplementary Material). Because CD-HIT and UCLUST use a different approach to clustering, they are not directly comparable and are included for reference only..

In HPC-CLUST, the largest fraction of computation time is spent calculating the pairwise sequence distances, the second largest in sorting the distances and the final clustering step is the fastest. HPC-CLUST can make use of multithreaded execution on multiple nodes and practically achieves optimal parallelization in the distance calculation step. Additional benchmarks are shown and discussed in the Supplementary Material.

### 3.2 Distributed clustering performance

Clustering the full dataset (833 013 unique sequences) to 97% identity threshold required a total of 2 h and 42 min on a compute cluster of 24 nodes with 8 cores each (192 total cores). Owing to parallelization, the distance and sorting computation took only 57 min (wall clock time), corresponding to >10 000 min CPU time. The remaining 1 h and 45 min (wall clock time) were spent collecting and clustering the distances. The combined total memory used for the distance matrix was 59.8 or 2.6 GB per node. The node on which the merging step was performed used a maximum of 4.9 GB of memory when doing single-, complete- and average-linkage clusterings in the same run

## 4 CONCLUSION

Clustering is often among the first steps when dealing with raw sequence data, and therefore needs to be as fast and as memory efficient as possible. The implementation of a distributed version of hierarchical clustering in HPC-CLUST makes it now possible to fully cluster a much larger number of sequences, essentially limited only by the number of available computing nodes.

## Supplementary Material

Supplementary Data
