# Supplementary material for: HPC-CLUST: distributed hierarchical clustering for large sets of nucleotide sequences
Source: Bioinformatics. 2013 Nov 9;30(2):287–8. doi: 10.1093/bioinformatics/btt657 (PMC3892691; doi:10.1093/bioinformatics/btt657)
Supplement: Supplementary Data [file supp_30_2_287__index.html]

HPC-CLUST: Distributed hierarchical clustering for very large sets of nucleotide sequences — HPC-CLUST: distributed hierarchical clustering for large sets of nucleotide sequences — HPC-CLUST: distributed hierarchical clustering for large sets of nucleotide sequences — Supplementary Data 

# HPC-CLUST: distributed hierarchical clustering for large sets of nucleotide sequences

## Supplementary Data

files

**Files in this Data Supplement:**

- Supplementary Data - pdf file
