# Supplementary material for: HPC-CLUST: distributed hierarchical clustering for large sets of nucleotide sequences
Source: Bioinformatics. 2013 Nov 9;30(2):287–8. doi: 10.1093/bioinformatics/btt657 (PMC3892691; doi:10.1093/bioinformatics/btt657)
Supplement: Supplementary Data [file supp_btt657_revised-supplementary-information.pdf]

Supplementary Information
Text S1

HPC-CLUST: Distributed hierarchical clustering for very large sets of nucleotide sequences

João F. Matias Rodrigues[1,*] and Christian von Mering[1,*]

[1]Institute of Molecular Life Sciences and Swiss Institute of Bioinformatics, University of Zurich, Zurich, Switzerland

October 19, 2013

*To whom correspondence should be addressed. Email: joao.rodrigues@imls.uzh.ch, mering@imls.uzh.ch tel. +41 44 635 3147

Supplementary Methods

We compared HPC-CLUST to previously existing clustering pipelines, by clustering identical sets of nucleotide sequences (we tested increasingly larger sets). Before running each program, the input file was read once with the linux utility "cat" to ensure that it would be cached prior to running the program. The following commands were used for clustering using a single thread, at 97% cluster identity threshold, and, when applicable, using single-linkage.

hpc-clust:

```
hpc-clust -ncpus 1 -sl true -t 0.97 -ofile bacteria-aligned bacteria-aligned.sto
```

mothur:

```
unique.seqs(fasta=bacteria-aligned.fasta);
dist.seqs(fasta=bacteria-aligned.unique.fasta,output=lt,processors=1,cutoff=0.97);
hcluster(phylip= bacteria-aligned.unique.phylip.dist,method=closest,cutoff=0.97);
```

cd-hit:

```
cd-hit-est -i bacteria-unaligned.fasta -o results.out -c 0.97 -g 1 -T 1
```

uclust:

```
usearch -threads 1 -cluster_fast bacteria-unaligned.fasta -id 0.97
       -uc results.out -maxaccepts 0 -maxrejects 0
```

Runtime and memory usage was monitored using a continuously running script that parsed the /proc/PID/status file for the process belonging to the program. The

memory usage of ESPRIT could not be measured because the method used here does not take into account memory usage by child processes, as such the memory usage was incorrectly being reported as being zero for ESPRIT since many of its clustering steps involve calling external programs that are run as child processes.

Supplementary Benchmarks

Alignment

The alignment times when using INFERNAL to align the sequences for HPC-CLUST and MOTHUR were on average 1.5 seconds per sequence, for sequences with lengths of roughly 1300 bp. The alignment times were added to the runtimes of HPC-CLUST and MOTHUR to make them comparable to other methods that perform the alignment internally. When considering the use of multiple cores, the alignment times were divided by the number of cores to reflect the fact that alignment jobs with INFERNAL can easily be split into parts and are thus trivially parallelized.

Multithreading

HPC-CLUST is capable of parallelizing the more expensive steps of the clustering process, resulting in a large decrease in wall clock runtime. Other clustering programs (although supporting multithreading), were found to differ in their ability to effectively use multiple threads on a single machine. Figure S1 shows the differences in runtime between clustering programs running on a single thread versus eight threads. Both HPC-CLUST and MOTHUR were capable of nearly optimal

parallelization, resulting in essentially eight times less wall clock runtime. On the other hand, UCLUST and CD-HIT did not appear to make much use of the multiple threads.

Clustering thresholds

The choice of clustering threshold can impact the runtime and memory usage of clustering programs. Decreasing the identity threshold forces HPC-CLUST to store more pairwise distances in the distance matrix calculation step, increasing the memory requirements. It also increases the number of distances to be sorted and clustered, resulting in longer runtime, although the increase is practically negligible compared to the sequence alignment and distance computation times. This trend is observed in Figure S2, which shows the dependence of runtime and memory on clustering threshold for the programs tested. MOTHUR, which also implements hierarchical clustering, behaves in a similar way, although distances are normally written to disk. The runtimes of UCLUST and CD-HIT, on the other hand, depend on the number of OTUs formed. UCLUST increases in runtime with increasing thresholds. This is likely due to the increase in the number of OTUs that need to be searched during the clustering process. Strikingly, CD-HIT exhibits the opposite trend, being faster when there are larger numbers of OTUs.

Linkage method

Figure S3 shows that the linkage method also has some impact on runtime and memory usage. HPC-CLUST is faster than MOTHUR in all cases, and it exhibits

little difference in runtime depending on the linkage clustering method. In terms of memory usage, HPC-CLUST requires roughly the same amount of memory in all three linkage types, with the most memory being used in average-linkage, less in complete-linkage, and the least in single-linkage clustering. MOTHUR tends to use more memory than HPC-CLUST, although for average-linkage, the trend at larger number of sequences shows HPC-CLUST increasing in memory requirements faster than MOTHUR.

Algorithms and implementation details

The general approach in hierarchical clustering algorithms is to start with a completely partitioned set of sequences (one sequence per cluster) and sequentially merge the two nearest clusters up to a predefined threshold. In the case of singleton clusters (i.e., clusters composed of a single sequence) the distance between clusters is the dissimilarity between the two sequences; but when comparing clusters with multiple elements, different cluster distance metrics can be used. In HPC-CLUST, we have implemented three metrics that define the distance between clusters, namely: single-linkage, i.e. the distance between clusters is the distance between the least dissimilar sequences; complete-linkage, i.e. the cluster distance is the distance between the two most dissimilar sequences; and average-linkage, i.e. the cluster distance is the average pairwise dissimilarity between the sequences in the two clusters.

There are many ways to implement hierarchical clustering algorithms, however our implementation has two benefits: 1) it allows the generation, in a single computation, of a merge log from which OTUs can be generated at any level down to a

clustering threshold given by the user, and 2) the distance matrix can be distributed over several computing nodes with minimal impact on clustering times.

We will begin by explaining the single-linkage implementation on a single computer, as it is the simplest and the explanation for the complete-linkage, average-linkage and distributed versions can be based upon it.

In single-linkage clustering, we begin by identifying any duplicate sequences and then compute the pairwise sequence distances between all unique sequences in the input dataset. The distances and indices of the sequences found above the given threshold are stored in an array and sorted from closest to most distant; we define a distance and an index as a distance element, and the array as the distance array. To implement the hierarchical clustering algorithm, we simply process each distance element in the distance array starting from the top (the most similar two sequences) and merge the clusters to which the sequence pairs belong to. This approach gives exactly the same result as if one would choose the closest two clusters at each clustering step. A proof for this is straightforward, in the initial case when all clusters are singletons, the distance between clusters is the pairwise distance between sequences, therefore the closest two clusters are exactly the closest two sequences. After a few clustering steps, some clusters will have more than one sequence, at this point the two closest clusters must have (using the single-linkage definition) the two most similar sequences, therefore they will also be the first unprocessed distance element at the top of the distance array. Note that when merging two clusters i and j with more than one sequence each, several distance elements will be encountered in the distance array (exactly $N_i N_j$, where N_i is the number of sequences in cluster i, and N_j is the

number of sequences in cluster j); a merge event (the step at which two clusters are considered merged) occurs only when the first distance element between sequences in the two clusters is encountered with subsequent distance elements between the already merged cluster pair being ignored.

To avoid having to rename the identity of all sequence indices in the whole distance array every time a merge event occurs, a vector is used that contains the identity of the cluster that each sequence belongs to; we refer to this as the cluster membership vector. For every distance processed, the cluster that each sequence belongs to is looked up in the cluster membership vector and when they are different, a merge event is recorded and the cluster identity of the sequences is updated in the vector.

In complete-linkage clustering, the process is very similar to single-linkage: the distances are also computed and sorted. However, in contrast to single-linkage, two clusters should only be merged when the *last* distance element between the two clusters is encountered in the distance array. In this manner, finding the two closest clusters is equivalent to finding the first distance element that completes the linkage between two clusters; a link being a distance element being processed in the distance array connecting a cluster pair. This approach therefore only requires the knowledge of how many distance elements ($C_{i,j}$) have been processed for each cluster pair. For speed and memory efficiency reasons, a hash table was chosen to store the processed distance element counts between clusters. This table is kept up-to-date by increasing the distance element count between two clusters every time a distance is processed linking them. Another requirement is that counts be updated in response to the

merging of two clusters. Consider for example three clusters i, j, and k, with sizes N_i, N_j, and N_k, respectively and let $C_{i,j}$, $C_{i,k}$, and $C_{j,k}$ be the distance element counts observed between the clusters so far. If the last distance element between i and j is processed such that $C_{i,j} = N_i N_j$ then the distance element counts between the new merged cluster ij and k will be $C_{ij,k} = C_{i,k} + C_{j,k}$. The efficiency of this implementation depends on how large the hash table becomes. In practice, the number of entries in the hash table never increases dramatically for four reasons: i) only clusters with at least one link are kept in the hash table, ii) clusters that are close enough to be linked ($C_{i,j} > 0$) eventually get merged, iii) when a merge event occurs, the number of entries will decrease by the number of links shared between the two merged clusters, and iv) as the clustering proceeds, fewer clusters exist and therefore fewer possible cluster pairs can be linked.

Average-linkage clustering is implemented in a similar fashion to complete-linkage but with a few modifications. In average-linkage, the distance between two clusters is only known once all distance elements have been processed; in this sense, a similar approach is used as in the complete-linkage implementation. One difference is that in addition to the count of distance elements ($C_{i,j}$) processed so far between clusters, the average distance between clusters ($A_{i,j}$) is also kept track of. Once all distance elements between two clusters have been processed, the cluster merge event is placed on a temporary merge list that is sorted with respect to the average distance of the merge event. The reason for this list is due to complete-linkage between clusters not being synonymous with average distance between clusters, i.e.: there can be an as yet incompletely linked ($C_{i,j} < N_i N_j$) cluster pair with a lower final average distance

(when complete) than an already completely linked cluster pair. The merge list therefore serves to keep track of the cluster merge order until sufficient information exists to be certain about the merge order. It is possible to be certain about a merge order up to a certain distance S by making use of two facts: 1) that the distances in the distance array are sorted and therefore no smaller distance will be found than the current distance (D) being processed, i.e.: D is the minimum sequence distance; and 2) the smallest possible final average distance for an incomplete cluster merge will be $S_{i,j} = \frac{C_{i,j}A_{i,j}+(N_iN_j-C_{i,j})D}{N_iN_j}$. In other words, $S_{i,j}$ for an incompletely linked merge represents the lower bound on the final average distance that two clusters would have if all of the remaining unprocessed sequence distances ($N_iN_j - C_{i,j}$) had the same distance as the current minimum sequence distance D at a given clustering step. As the clustering proceeds, every time the minimum distance D increases, the lower bound $S = \min_{\forall\ (i,j)} S_{i,j}$ is recomputed for all incomplete cluster pairs. At this point, all completely-linked merge events not yet processed with average distance smaller than S can be merged since it is guaranteed that no incompletely-linked merge events exist with smaller final average distance than S.

In the distributed implementation of HPC-CLUST, the actual clustering is performed on a single node, while the distance computation is distributed across all other nodes. Once all nodes have finished computing their parts of the distance matrix and have sorted the distances, they begin transferring their distance elements to the clustering node where distance elements are further sorted and processed sequentially in the usual manner as described above for the single computer case. It is worth noting that even in the distributed version of HPC-CLUST, the distance

matrix is never written to disk and therefore any bottlenecks associated with disk access are circumvented.

For memory and computational efficiency, HPC-CLUST compresses the sequences such that each nucleotide uses only 4 bits instead of the usual 8 bits. Other improvements have been made in the distance calculation by making use of 64bit variables and therefore performing the comparison of 16 nucleotides at a time.

For the distance computation, four distance functions were implemented in HPC-CLUST, these are: gap, nogap, nogapsingle, and tamura. The "gap" distance function counts gap-gap as matches, and gap-nucleotide as mismatches, this brings sequences closer if they share the same gaps in the alignment. The "nogap" distance function ignores gap-gap cases, while gap-nucleotide are considered mismatches. The "nogapsingle" ignores gap-gap and counts a row of consecutive gap-nucleotide as a single mismatch. Finally, the "tamura" distance function implements a corrected distance that accounts for differences in transition/transversion rates and G+C-content biases as described in Tamura (1992).

Validation of HPC-CLUST clustering results

We performed a validation of the clustering results from HPC-CLUST by comparing them to the results obtained from MOTHUR on the same dataset. Here we used a set of 8631 sequences that comprise the 16S rRNA sequences extracted from the Refseq genome database of NCBI. Two validations were performed, in the first case we verified the results of the distance calculation step by plotting the distances computed by HPC-CLUST vs. those computed by MOTHUR (Figure S4). The

computed distances are very similar but not always identical, due to differences in the distance calculation approaches taken in HPC-CLUST and MOTHUR. In the second case, we verify the results of the clustering part by using the same distances (as calculated by HPC-CLUST) in both programs. In Figure S5, we show that the OTU counts for each threshold are very similar (and actually perfectly equal in the case of single-linkage clustering). Finally, we compared the OTU set resulting from HPC-CLUST and MOTHUR at 98% clustering for the three linkages using the Rand Index (Rand , 1971). The Rand Index (RI) provides a measure of the similarity of two partitions, ranging from 1 when the partitions are identical and 0 when the partitions are completely unrelated. The results from HPC-CLUST and MOTHUR were identical (single-linkage RI: 1.0) or nearly identical (average-linkage RI: 0.99, complete-linkage RI: 0.99). When clustering using complete-linkage and average-linkage methods, the final OTU set may vary for the same dataset and clustering threshold, due to the possibility that several merge events occur at the same threshold and therefore no consistent order for these merges is defined. This is not a limitation that affects only HPC-CLUST but any other hierarchical clustering implementations of average- and complete-linkage.

References

Rand, W. M. (1971). Objective Criteria for the Evaluation of Clustering Methods. *J. Am. Stat. Assoc.*, **66**(336):846-850.

Tamura K. (1992). Estimation of the number of nucleotide substitutions when there are strong transition-transversion and G+C-content biases. *Mol. Biol. Evol.*, **9**(4):678687.

Supplementary Figures

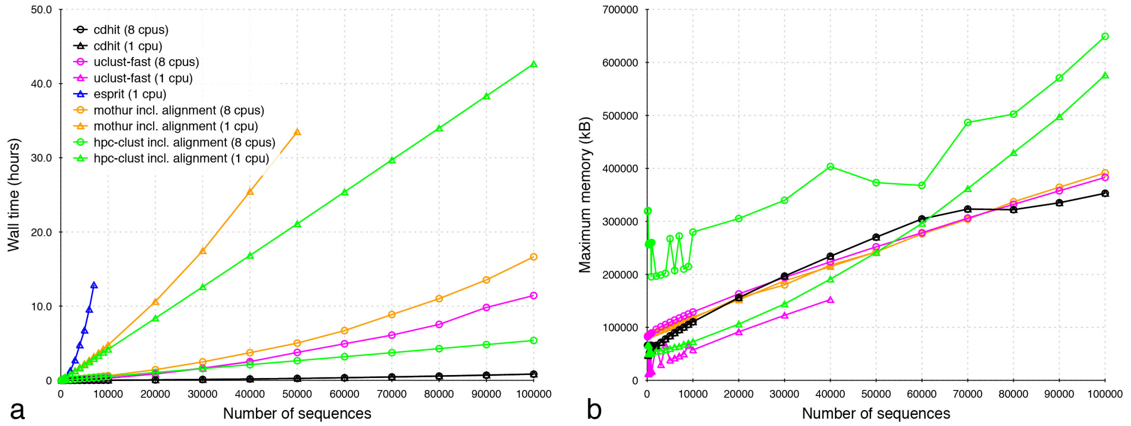

Figure S1: a) Wall clock runtime, and b) memory usage, depending on number of threads. Clustering to 98% threshold (complete-linkage clustering, when applicable).

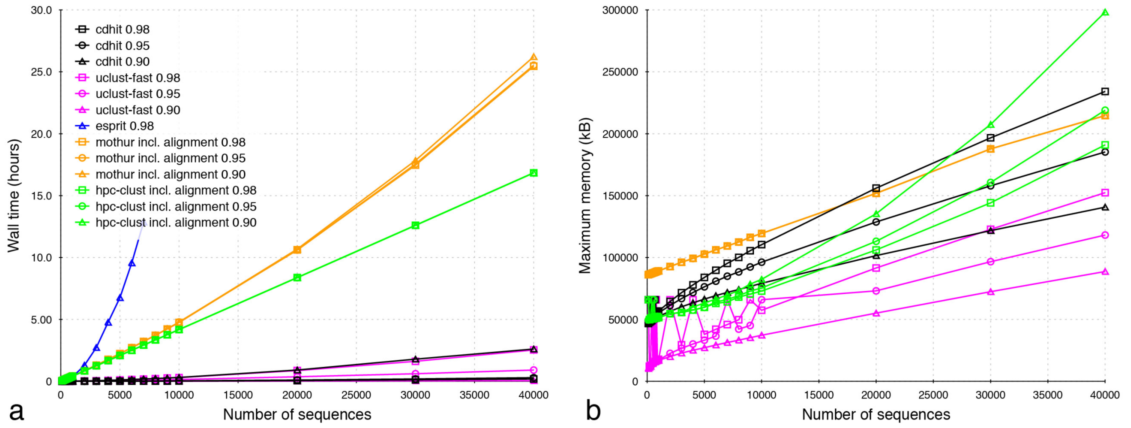

Figure S2: a) Runtime, and b) memory usage, depending on clustering thresholds. Clustering on a single CPU (complete-linkage clustering, when applicable).

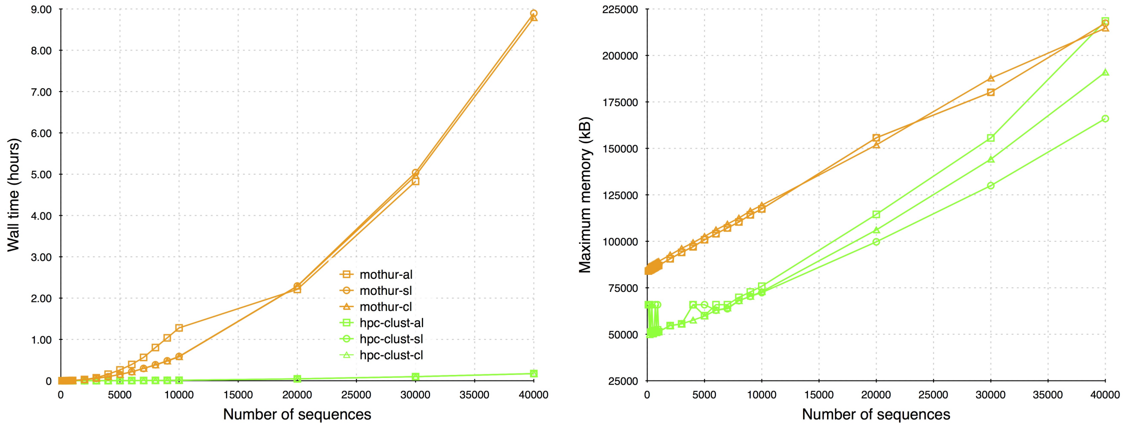

Figure S3: a) Runtime without sequence alignment time for HPC-CLUST and MOTHUR, and b) memory usage dependence on different clustering linkage types: (cl) complete-linkage, (sl) single-linkage, and (al) average-linkage. Clustering to 98% threshold on a single CPU.

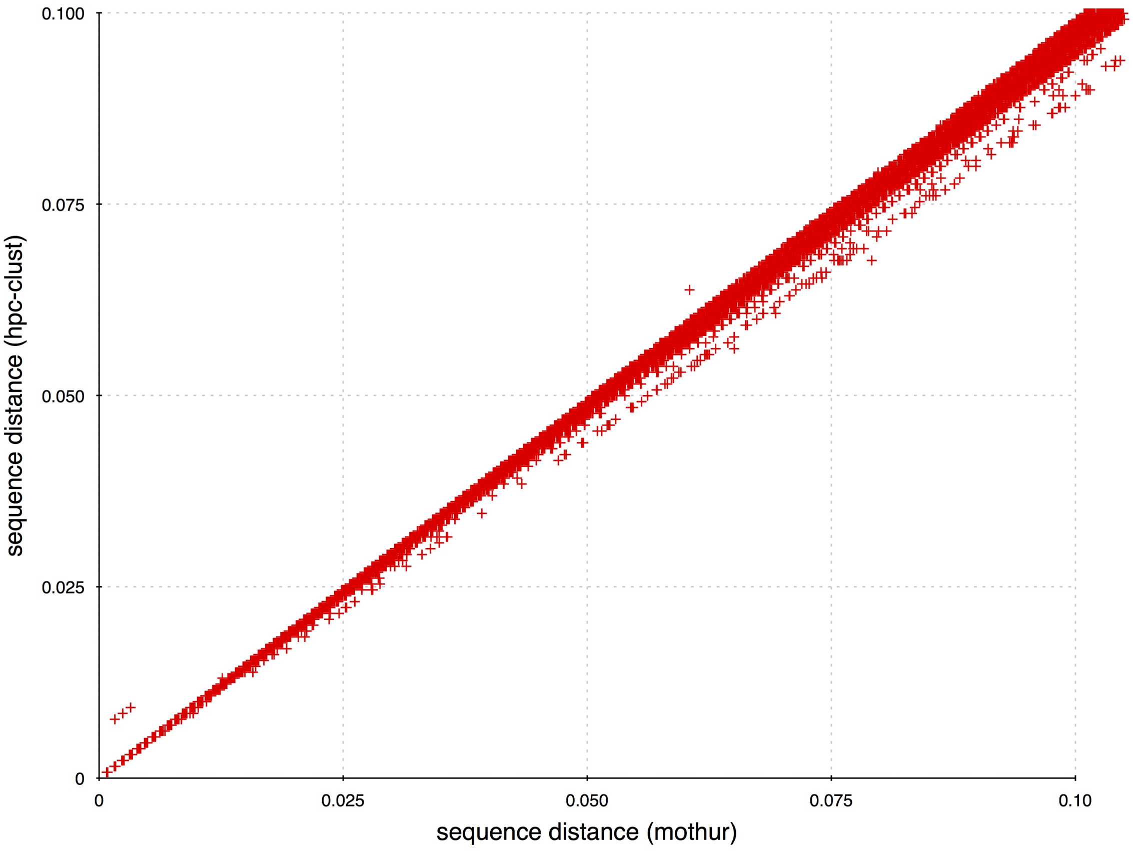

Figure S4: Comparison of distance calculation between HPC-CLUST and MOTHUR.

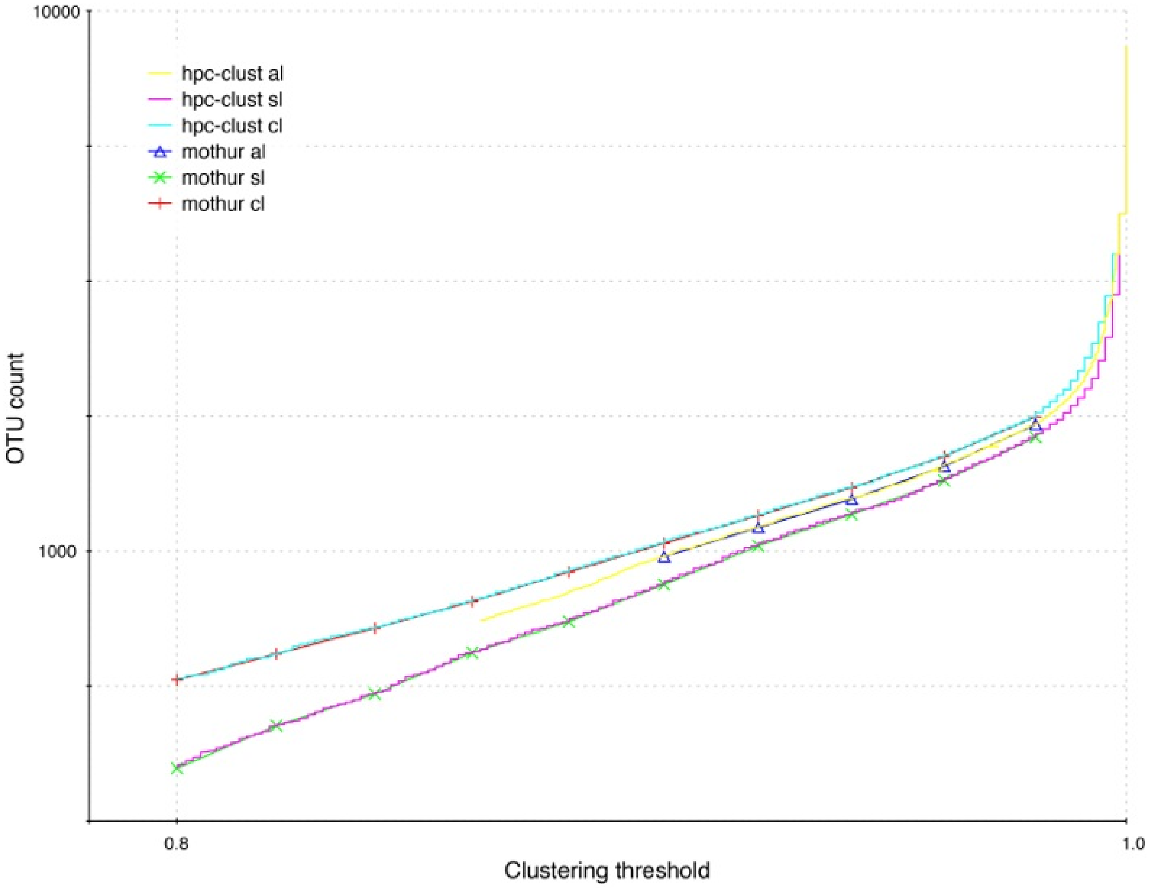

Figure S5: Comparison of clustering results between HPC-CLUST and MOTHUR using the same distance array in both programs for different clustering linkage types: (cl) complete-linkage, (sl) single-linkage, and (al) average-linkage.
